# Transcription Regulation of *Tceal7* by the Triple Complex of Mef2c, Creb1 and Myod

**DOI:** 10.3390/biology11030446

**Published:** 2022-03-16

**Authors:** Zhenzhen Xiong, Mengni Wang, Shanshan You, Xiaoyan Chen, Jiangguo Lin, Jianhua Wu, Xiaozhong Shi

**Affiliations:** 1 School of Biology and Biological Engineering, South China University of Technology, Guangzhou 510006, China; zhenzhenxiong95@163.com (Z.X.); wangmengni20@163.com (M.W.); shanshan7936@163.com (S.Y.); xiaoyanchen01@hotmail.com (X.C.); wujianhua@scut.edu.cn (J.W.); 2 Research Department of Medical Sciences, Guangdong Provincial People’s Hospital, Guangdong Academy of Medical Sciences, Guangzhou 510080, China; linjiangguo@gdph.org.cn; 3 Department of Emergency Medicine, Guangdong Provincial People’s Hospital, Guangdong Academy of Medical Sciences, Guangzhou 510080, China

**Keywords:** *Tceal7*, Mef2c, Creb1, Myod, skeletal muscle, myogenesis

## Abstract

**Simple Summary:**

We have previously reported a striated muscle-specific gene during embryogenesis, *Tceal7*. Our studies have characterized the 0.7 kb promoter of the *Tceal7* gene, which harbors important E-box motifs driving the LacZ reporter in the myogenic lineage. However, the underlying mechanism regulating the dynamic expression of *Tceal7* during skeletal muscle regeneration is still elusive. In the present work, we have defined a cluster of Mef2#3–CRE#3–E#4 motifs through bioinformatic analysis and transcription assays. Our studies suggested that the triple complex of Mef2c, Creb1 and Myod binds to the Mef2#3–CRE#3–E#4 cluster region, therefore driving the dynamic expression of *Tceal7* during skeletal muscle regeneration. The novel mechanism may throw new light on understanding transcription regulation in skeletal muscle myogenesis.

**Abstract:**

*Tceal7* has been identified as a direct, downstream target gene of MRF in the skeletal muscle. The overexpression of *Tceal7* represses myogenic proliferation and promotes cell differentiation. Previous studies have defined the 0.7 kb upstream fragment of the *Tceal7* gene. In the present study, we have further determined two clusters of transcription factor-binding motifs in the 0.7 kb promoter: CRE#2–E#1–CRE#1 in the proximal region and Mef2#3–CRE#3–E#4 in the distal region. Utilizing transcription assays, we have also shown that the reporter containing the Mef2#3–CRE#3–E#4 motifs is synergistically transactivated by Mef2c and Creb1. Further studies have mapped out the protein–protein interaction between Mef2c and Creb1. In summary, our present studies support the notion that the triple complex of Mef2c, Creb1 and Myod interacts with the Mef2#3–CRE#3–E#4 motifs in the distal region of the *Tceal7* promoter, thereby driving *Tceal7* expression during skeletal muscle development and regeneration.

## 1. Introduction

The process of skeletal muscle terminal differentiation involves a precisely orchestrated series of events, including cell cycle exit, myoblast fusion and formation of multinucleated myotubes, which are cooperatively driven by a variety of ubiquitous and muscle-specific transcription factors [1]. Myod, as a master regulator in the skeletal muscle lineage, belongs to the family of basic helix–loop–helix (bHLH) transcription factors, which can form heterologous complexes with E2 family proteins before binding to the canonical DNA sequence (CANNTG, referred to as the E-box) to transactivate its downstream target genes, such as Desmin, MCK, *Tceal7*, Kbtbd5 and Rb1 [2,3,4,5,6]. Myod^−/−^ mice have no apparent defect in their skeletal muscle development due to the functional compensation of Myf5 [7]. However, the loss of Myod has results in the conversion of satellite cell-derived primary myoblasts into brown adipocytes by upregulating Prdm16, while a high expression of Myod in brown preadipocytes prevents brown adipogenesis [8]. Recent studies have also revealed that Myod regulates skeletal muscle differentiation by its downstream miRNA targets [9]. For example, Myod can directly activate MiR206 to promote myogenic differentiation by inhibiting Pax3 and Pax7 [10,11]. Recent studies have also reported that Myod plays a pivotal role in superenhancer assembly and activation, as well as 3D genome structure organization in muscle cells [12,13]. Myod knockout or knockdown cells generated by CRISPR-Cas9 or siRNAs present a lower expression level of myotube eRNA (enhancer RNA), but moderately increase myoblast eRNA [14]. It has also been demonstrated that Myod executes its function in skeletal muscle myogenesis through a number of cofactors, such as Mef2c and Creb1 [6,15].

Myocyte enhancer factor 2 (Mef2) is a member of the MADS-box family of transcription factors, which consists of Mef2a, Mef2b, Mef2c and Mef2d in vertebrates [15]. Mef2 proteins share highly conserved MADS-box and MEF2 domains, which recognize the Mef2 motif, characterized as an A/T-rich sequence, YTA(A/T)_4_TAR, in the regulatory region of downstream genes through high-affinity DNA binding and dimerization [15,16,17]. Mef2c is detectable in the myotome at E9.0 during embryogenesis, earlier than Mef2a and Mef2d [18]. The complete deletion of Mef2c causes embryonic lethality on account of severe cardiovascular abnormalities in mice [19]. Conditional knockout Mef2c in the skeletal muscle results in birth defects due to a severely disorganized myofiber [20]. In contrast, the loss of Mef2a or Mef2d does not affect the skeletal muscle development in mice [16]. Moreover, Mef2c has been identified as a direct downstream target of Myod, and forms a transcription complex with Myod as positive feedback to regulate muscle-specific gene expression, thereby inducing myogenic differentiation [21,22].

The cAMP-responsive element-binding protein (Creb1) belongs to a subgroup of bZIP transcription factors that includes cAMP-responsive element modulators (CREMs) and activating transcription factor 1 (ATF1) [23,24]. Creb1 specifically binds to the conservative CRE elements harboring a full-site TGACGTCA or a half-site CGTCA/TGAGC as a homodimer or heterodimer [25,26,27]. The phosphorylation of Creb1 at Ser133 is significant for elevating its transcription activity, which promotes the recruitment of coactivator p300/CBP [28,29]. However, the mutation of Ser133 fails to completely abolish the transcription activity of Creb1 due to a constitutive activity provided by the Q2 domain of Creb1 [30]. Creb1^−/−^ mice die immediately after birth due to respiratory defects, and have impaired T-cell development and reduced corpus callosum and anterior junction in the brain [31]. Recent evidences have suggested that Creb1 is also a player in skeletal muscle development, growth and survival [32,33,34]. The reduction in Creb1 activity in adult mice causes muscular dystrophy and slows down the process of skeletal muscle regeneration [32,33]. In addition, our recent studies have suggested that Creb1 may be a critical transcription factor regulating dynamic gene expression [35]. In summary, Creb1 plays an essential role in cellular function through its interactions with multiple transcription factors, such as Myod, Mesp1 and AP1 [27,36,37].

Our previous work has characterized the specific spatiotemporal pattern of *Tceal7* gene expression and its 0.7 kb promoter, which drives the expression of the LacZ reporter in skeletal muscle lineage through the five E-box motifs transactivated by Myod [4]. In the present work, we aim to investigate the regulatory mechanism of the dynamic expression of *Tceal7* during skeletal muscle regeneration. Herein, our analysis has revealed two clusters of motifs: CRE#2–E#1–CRE#1 in the proximal region and Mef2#3–CRE#3–E#4 in the distal region. We have also examined the transactivation of *Tceal7* by the cofactors of Myod: Mef2c and Creb1. Interestingly, we have observed a synergistic transactivation between Mef2c and Creb1 with the Reporter II, which prompts us to examine the protein–protein interaction between Mef2c and Creb1. In summary, our present studies support the notion that the triple complex of Mef2c, Creb1 and Myod bind to the cluster of Mef2#3–CRE#3–E#4 motifs in the distal region of the *Tceal7* promoter, thereby transactivating *Tceal7* expression during skeletal muscle development and regeneration.

## 2. Materials and Methods

### 2.1. Plasmid Construction

The manipulation of DNA and RNA was performed using the Genomic DNA Extraction Kit, EASYspin plus RNA Extraction Kit and TRUEscript 1st Strand cDNA Synthesis Kit (Aidlab Biotechnologies, Beijing, China), respectively. Wild-type (Wt) and mutant (Mut) forms of the *Tceal7* 0.7 kb promoter fused to luciferase reporters were generated by routine PCR or site-specific mutagenesis PCR. Reporter I and Reporter II were constructed by two pairs of complementary primers using PCR. A pGL3-3×(Mef2#3) luciferase reporter was purchased from Miaoling Plasmid Sharing Platform (Wuhan, China). *Mef2c* (NM_001170537.1), *Creb1* (NM_133828.2) and *Myod* (M18779.1) were subcloned into the expression vectors: pcDNA-HA, pcDNA-3×HA and pCS2-6×Myc. Creb1(1−327), encoded by NM_133828.2, is a variant of Creb1(1−324) with a partial Q1 deletion due to exon skipping, both of which are transcriptionally active. Residues at S119 and Y134 of Creb1(1−327) are equivalent to those at S133 and Y120 of Creb1(1–324), respectively. A mutation of the residue Y120F resulted in the constitutive activity of Creb1. *Creb1 Y120F* and *Creb1 S119A* were subcloned into pcDNA-HA and pcDNA-3×HA vectors through mutagenesis PCR [6,26].

### 2.2. Cell Culture and Cell Transfection

C2C12 myoblasts and NIH3T3 cells were purchased from ATCC (American Type Culture Collection, Manassas, VA, USA) and CAS (Chinese Academy of Sciences, Beijing, China), respectively. HEK293T cells were kindly provided by Prof. Jianhua Wu (South China University of Technology, Guangzhou, China). The C2C12 myoblasts, NIH3T3 and HEK293T cells were all maintained in high-glucose DMEM, supplemented with 10% FBS (HyClone, Logan, UT, USA), 2 mM L-Glutamine, 100 U/mL penicillin and 100 μg/mL streptomycin (Transgen Biotech, Beijing, China) (growth medium, GM) in a 5% CO_2_ incubator at 37 °C. The C2C12 myoblasts (passage 7~10) were transfected using Via-FectTM (Promega, Madison, WI, USA), while the NIH3T3 and HEK293T cells were transfected with TransIntroTM EL Transfection Reagent (Transgen Biotech, Beijing, China). For C2C12 myoblast differentiation, the cell-cultured medium was switched to DMEM containing 2% horse serum, 100 U/mL penicillin and 100 μg/mL streptomycin (differentiation medium, DM) once the C2C12 myoblasts became confluent.

### 2.3. Transcription Assays

The C2C12 myoblasts and NIH3T3 cells were seeded into 6-well plates, and the HEK293T cells into 12-well plates, the day before transfection. These cells were then transfected with the following plasmids: 400 ng reporter plasmid (Wt, Mef2-Mut, pGL3-3×Mef2#3, Reporter I or Reporter II), 10 ng internal control (pRL-CMV) and 200–1000 ng expression plasmids (Mef2c, Creb1 or Myod). The total DNA amount was normalized with the empty expression vector pcDNA3.1. The transfected cells were collected and lysed 24 h after transfection, followed by an analysis with the Dual-Luciferase^®^ Reporter Assay System (Promega, Madison, WI, USA). All fold changes in the firefly luciferase activity were normalized to that of the Renilla luciferase [27].

To measure the activity of the *Tceal7* promoter or its mutant in myotubes, 1 × 10^5^ C2C12 myoblasts were seeded into 6-well plates on the day before transfection. Wild-type and mutant reporters (400 ng) with a pRL-CMV plasmid (10 ng) were cotransfected into the C2C12 myoblasts, with a mass ratio of 40:1. After 24 h, the growth medium was replaced with the differentiation medium for 48 h. At harvest time, the myotubes were washed twice with PBS, lysed in a precooled lysis buffer (PBS supplemented with 20% glycerol, 0.1% Triton, 1× complete protease inhibitor (Roche, Basel, Switzerland) and 1 mM dithiothreitol) on ice for 10–15 min; then, they were utilized for luciferase assay after the freeze–thaw cycle. The relative activity of the Wt reporter was set as 100% [4].

### 2.4. Western Blot and Coimmunoprecipitation Assays

The HEK293T cells were transfected with 3×HA-Creb1 or its deletional mutants (3 µg) and 6×Myc-Mef2c or its deletion expression plasmids (3 µg) for 24 h. The cells were washed with PBS and lysed in a lysis buffer (50 mM Tris pH 7.5, 150 mM NaCl, 5 mM EDTA, 0.1% Triton X-100, 1 mM dithiothreitol and 1× complete protease inhibitor). The total cell lysate was prepared by centrifuge at 14,000× *g* at 4 °C for 15 min. Western blot and coimmunoprecipitation assays were performed as previously reported [38]. The primary antibodies used included: rat monoclonal anti-HA antibody (Clone BMG-3F10, Roche, Switzerland), rat IgG control (sc-2026, Santa Cruz Biotechnology, Santa Cruz, CA, USA) and rabbit polyclonal antibody (#2272, Cell Signaling Technology, Danvers, MA, USA). The secondary antibodies used included: goat anti-rat IgG (#7077, Cell Signaling Technology, Danvers, MA, USA) and goat anti-rabbit IgG (#7074, Cell Signaling Technology, Danvers, MA, USA). The signal was detected by using a high-sensitivity substrate (WBKLS0050, Millipore, Billerica, MA, USA) in a chemiluminescence system.

### 2.5. ChIP Assays

The C2C12 myoblasts were transfected with an HA-Mef2c or HA-Creb1 expression plasmid (8 µg) and, 24 h later, switched to the differentiation medium for 48 h. The differentiated myotubes were then washed twice with PBS, crosslinked with 1% formaldehyde for 10 min at room temperature and quenched using 0.125 M Glycine. The myotubes were washed with precooled PBS again and collected for chromatin preparation. The following procedures were performed according to manufacturer’s protocol for the ChIP Assay Kit (Beyotime, Shanghai, China). Briefly, the cells were suspended in an SDS lysis buffer containing PIC and sonicated using the Bioruptor Plus (30 s on, 30 s off, 45 cycles). The chromatin was diluted with ChIP-dilution buffer and preincubated with 70 μL protein A + G agarose for 1 h at 4 °C. The supernatant was then incubated with an anti-HA antibody (C29F4, Cell Signaling Technology, Danvers, MA, USA) or a normal rabbit IgG (AB-105-C, R&D systems, Minneapolis, MN, USA) control, overnight at 4 °C. On the next day, each ChIP reaction was supplied with 40 μL protein A + G agarose and incubated for another 2 h at 4 °C. The agarose beads were collected by centrifuge at 1000× *g* at 4 °C for 1 min and washed as follows: twice with a ChIP-dilution buffer, once with a low-salt-wash buffer, once with a high-salt-wash buffer, once with a LiCl wash buffer and twice with a TE buffer. The immunoprecipitation complex was eluted twice with an elution buffer (1% SDS, 0.1 M NaHCO_3_) and incubated with NaCl and RNase A for reverse crosslinking at 65 °C for 6–16 h. The immunoprecipitated DNA was precipitated with ethanol and treated with Proteinase K at 45 °C for 1 h, followed by purification according to the manufacturer’s protocol for the DNA purification and recovery Kit (Aidlab Biotechnologies, China). Finally, it was resuspended in an elution buffer. A routine PCR was done with specific primers flanking the Mef2#3 or CRE#3 motif [4].

### 2.6. Statistics

All data represent the mean ± S.D. of at least three independent replicates. Statistical significance was assayed by one-way ANOVA for multiple comparisons with a Turkey’s test using a GraphPad prism. * *p* < 0.05; ** *p* < 0.01; *** *p* < 0.001; **** *p* < 0.0001; ns, not significant.

## 3. Results

### 3.1. The Functional Role of Mef2 and CRE Motifs in Tceal7 Promoter

**Figure 1:**
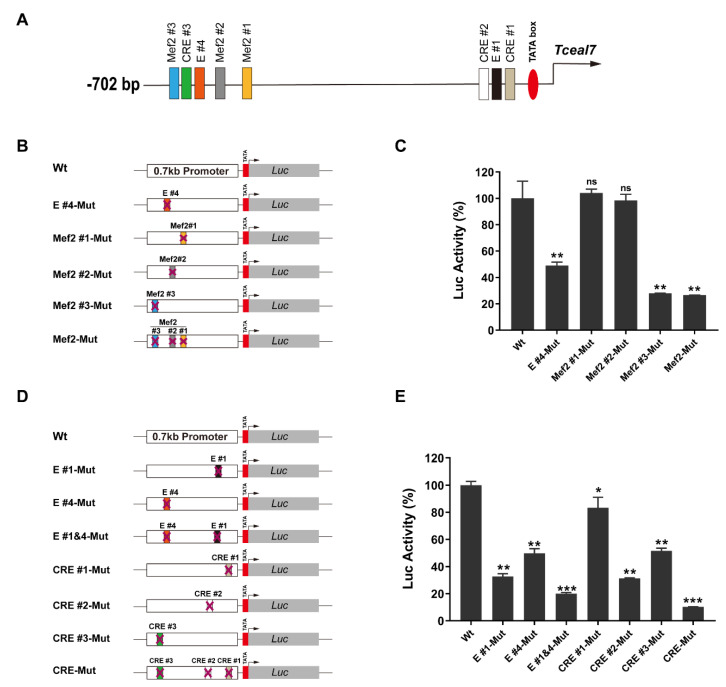
Functional role of Mef2 and CRE motifs in the *Tceal7* promoter: (**A**) Schematic illustration of the distribution of transcription factor-binding motifs in the *Tceal7* promoter. Three Mef2 motifs, three CRE motifs, two E-boxes and a TATA box are clustered into two regions: TATA box, CRE#1, E#1 and CRE#2 in the proximal region; Mef2#1, Mef2#2, E#4, CRE#3 and Mef2#3 in the distal region. (**B**) Schematic diagram of the luciferase reporter of the *Tceal7* promoter containing the mutation of the E#4 or Mef2 motif. Wt, wild-type reporter; Mef2#1-Mut, Mef2#1-mutant; Mef2#2-Mut, Mef2#2-mutant; Mef2 #3-Mut, Mef2#3-mutant; Mef2-Mut, mutation of Mef2#1, #2 and #3 motifs. (**C**) Mutation of Mef2#3 instead of Mef2#1 or Mef2#2 results in a decrease in the promoter activity (28% of that of the wild-type reporter) in the C2C12 myotubes. Additional mutation of Mef2#1 and Mef2#2 does not further reduce the promoter activity. Mutation of E#4 motif is utilized as an internal control. (**D**) Schematic diagram of the luciferase reporter of the *Tceal7* promoter harboring E-box or CRE motif mutation. CRE-Mut, mutation of CRE#1, #2 and #3 motifs; E#1&4-Mut, mutation of E#1 and E#4 motifs. (**E**) Each individual CRE motif mutant is accompanied by decreased activity in the C2C12 myotubes: CRE #1-Mut, 83%; CRE #2-Mut, 31%; and CRE #3-Mut, 52%. The promoter activity of CRE-Mut is further downregulated to 10% with an E-box mutant reporter as the reference. All Data represent the mean ± SD of three independent replicates. * *p* < 0.05; ** *p* < 0.01; *** *p* < 0.001; ns, not significant. The comparisons are analyzed between the Wt group and the Mut group.

Gene expression under a complicated physiological environment is generally regulated by multiple transcription factors, rather than simply one single factor [39,40]. Our previous studies have revealed that the expression of *Tceal7* in skeletal muscle is transactivated by Myod through the E#1 or E#4 box [4]. However, the mechanism driving its dynamic expression pattern during skeletal muscle regeneration remains a mystery. Herein, we have further analyzed the 0.7 kb promoter sequence to gain more insight into the transcriptional regulation of *Tceal7*. A bioinformatics analysis has revealed that there are three Mef2 motifs (Mef2#1–#3) and three CRE motifs (CRE#1–#3), which are distributed into two cluster regions around E#1 and E#4: the proximal region (CRE#1, E#1 and CRE#2) and the distal region (Mef2#1, Mef2#2, E#4, CRE#3 and Mef2#3), respectively (Table 1 and Figure 1A). Mutations of E#1 and E#4 have been examined in our previous study. Herein, the consensus sequences of these Mef2 and CRE motifs have been mutated to evaluate the functional role of each motif (Table 1). As shown in Figure 1B,C, a mutation of the Mef2#1 or the Mef2#2 motif has no effect on its activity. However, a mutation of the Mef2#3 motif results in decreased activity, almost the same as a mutation of the three Mef2 motifs: 28% of the Wt reporter, which is lower than that of the E#4-Mut. The data indicate that the Mef2#3 motif is the most significant among the three Mef2 motifs. Utilizing a similar strategy, the mutation of the CRE motifs has been constructed and assessed in the myotubes (Figure 1D). As shown in Figure 1E, the mutation of each CRE motif is associated with reduced activity: CRE#1-Mut (83%), CRE#2-Mut (31%) and CRE#3-Mut (52%). Interestingly, the reporter activity of the CRE-Mut is dramatically downregulated to 10% of the wild-type, indicating that all of the CRE motifs are necessary for the maximum activity of the *Tceal7* promoter. The reliability of these studies has been verified with the E#1-Mut, E#4-Mut and E#1&4 -Mut as internal controls. Collectively, our data support the notion that the Mef2#3 and the three CRE motifs are required for the transcription activity of the *Tceal7* promoter.

### 3.2. Transactivation of Tceal7 by Mef2c in the Distal Region

**Figure 2:**
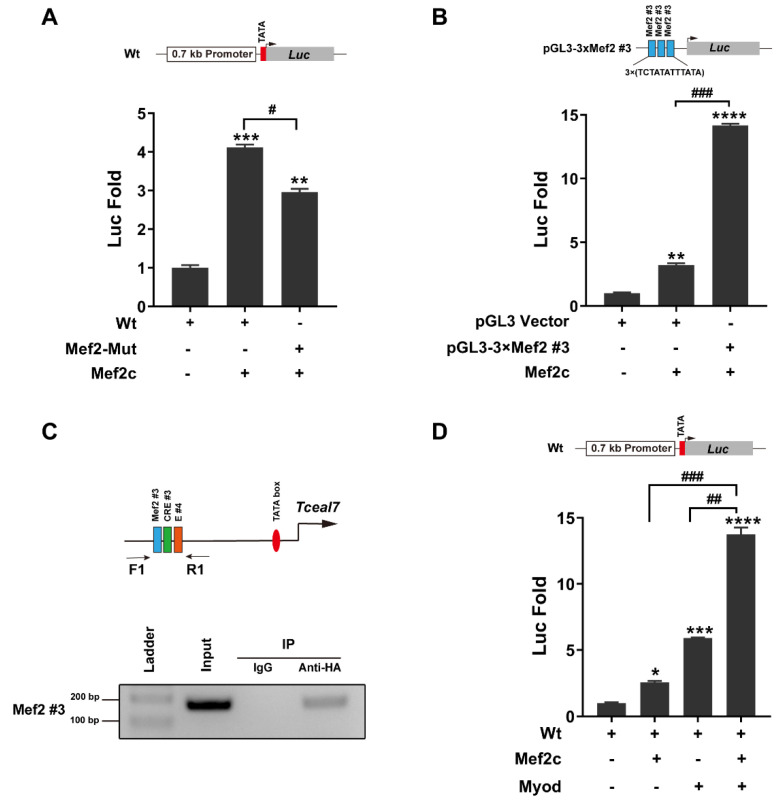
Transactivation of *Tceal7* by Mef2c. (**A**) The *Tceal7* 0.7 kb promoter reporter (Wt or Mef2-Mut) and Mef2c were cotransfected into C2C12 cells, and analyzed with a dual-luciferase assay system. The Wt reporter is transactivated by Mef2c up to four-fold, while the transactivation of the Mef2-Mut reporter is attenuated to three-fold. ** *p* < 0.01 and *** *p* < 0.001, comparison with the first group (Wt reporter). ^#^ *p* < 0.05. (**B**) Top panel, schematic diagram of the pGL3-3×Mef2 #3 luciferase reporter, which harbors a multimerized (3×) Mef2 #3 motif of the *Tceal7* promoter. The pGL3-3×Mef2 #3 luciferase reporter is transactivated by Mef2c up to 14-fold, while the transactivation of the pGL3 vector is around 3-fold in C2C12 cells. ** *p* < 0.01 and **** *p* < 0.001, comparison with first group (pGL3 vector). ^###^ *p* < 0.001. (**C**) Upper panel, ChIP–PCR primers (F1 and R1) are designed to amplify the Mef2#3 region specifically. The Mef2#3 region is enriched in the ChIP product with rabbit anti-HA antibodies, specifically, but not the control IgG. F1, forward primer; R1, reverse primer; input, total DNA product before immunoprecipitation. (**D**) Transactivation of the Wt reporter by Mef2c and Myod reaches up to 13.8-fold, exceeding that by Mef2c (2.6-fold) or Myod (5.9-fold) alone in NIH3T3 cells. * *p* < 0.05; *** *p* < 0.001 and **** *p* < 0.0001, comparison with first group (Wt reporter). ^##^ *p* < 0.01 and ^###^ *p* < 0.001. All Data represent mean ± SD of three independent replicates.

It has been reported that Mef2c can collaborate with Myod to transactivate the downstream targets during myogenic differentiation [22]. Our above studies have demonstrated that the Mef2 motifs in the promoter of *Tceal7* play an important role in its expression. Herein, Mef2c can transactivate the Wt reporter up to 4-fold, while the activation effect of the Mef2-Mut reporter is downregulated to 3-fold (Figure 2A). To confirm the role of the Mef2#3 motif in the transactivation by Mef2c, a reporter harboring a multimerized (3×) Mef2#3 motif was constructed and analyzed. As shown in Figure 2B, Mef2c is able to transactivate the pGL3-3×(Mef2#3) reporter up to 14-fold, dramatically exceeding that of the vector control. Further, a ChIP assay has been performed to verify the interaction between Mef2c and the Mef2#3 motif in the C2C12 myotubes (Figure 2C and Figure S1). Taken together, these studies have demonstrated the important role of Mef2#3 in the transcriptional regulation of *Tceal7* expression during myogenic differentiation. The adjacent location of Mef2#3 and E#4 has prompted us to examine the synergistic transactivation of *Tceal7* by Mef2c and Myod. As shown in Figure 2D, the Wt reporter is transactivated up to 13.8-fold in the presence of both Mef2c and Myod, much higher than Mef2c or Myod alone.

### 3.3. Myod and Creb1 Transactivate Tceal7 Expression through the Distal Region

**Figure 3:**
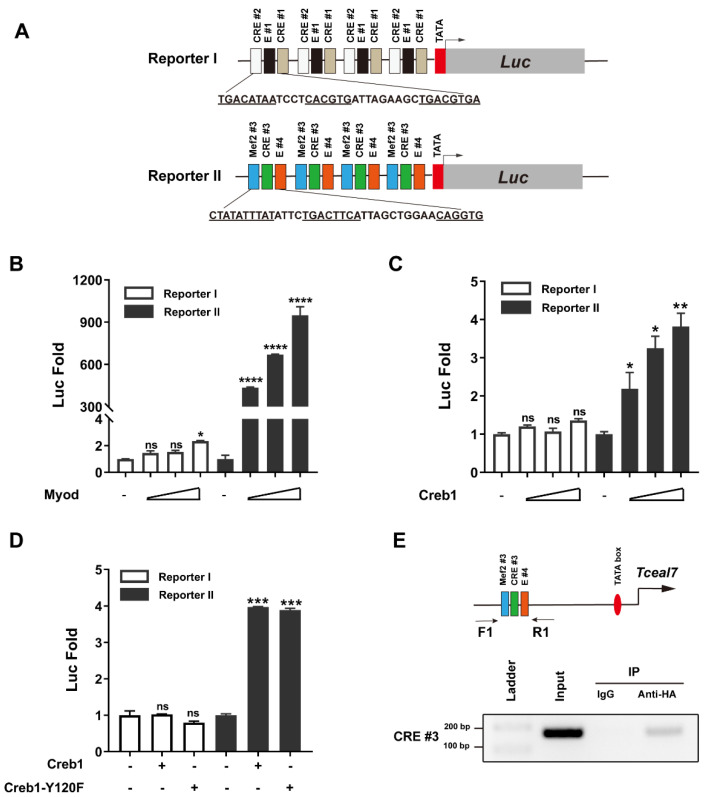
Transactivation of *Tceal7* by Myod and Creb1. (**A**) Schematic illustration of Reporter I and Reporter II. Reporter I and Reporter II contain multimerized sequences from the *Tceal7* promoter in a tandem manner: 4×(CRE#2–E#1–CRE#1) and 4×(Mef2#3–CRE#3–E#4), respectively. (**B**) Reporter I or Reporter II (400 ng) and Myod (200 ng, 400 ng and 600 ng) were cotransfected into the HEK293T cells, and analyzed with a dual-luciferase assay system. Myod can transactivate Reporter II up to 949-fold in a dose-dependent manner, while its transactivation of Reporter I is only 2.5-fold in HEK293T cells. * *p* < 0.05; **** *p* < 0.0001 and ns, not significant, comparison with first group (Reporter I or Reporter II). (**C**) Reporter I or Reporter II (400 ng) and Creb1 (200 ng, 400 ng and 600 ng) were cotransfected into the HEK293T cells, and analyzed with a dual-luciferase assay system. Creb1 can transactivate Reporter II up to 4-fold, but not Reporter I in HEK293T cells. * *p* < 0.05; ** *p* < 0.01 and ns, not significant, comparison with first group (Reporter I or Reporter II). (**D**) Creb1-Y120F or the wild-type Creb1 can transactivate Reporter II up to 4-fold, but not Reporter I in HEK293T cells. *** *p* < 0.001 and ns, not significant, comparison with first group (Reporter I or Reporter II). (**E**) Upper panel, ChIP primer design as in Figure 2D. Creb1 binds to the CRE #3 motif within the promoter of *Tceal7* in C2C12 myotubes. All data are shown as mean ± S.D from three replicates.

Creb1 is an important transcription factor involved in a variety of cellular processes during development; it directly transactivates multiple downstream target genes, such as Etv2, Col24α1, JHDM2a and Fndc5 [27,37,41,42]. Given that any mutation of the three CRE motifs in the *Tceal7* promoter results in activity reduction, two reporters (Reporter I and Reporter II) have been constructed by placing four repetitions of the two clusters (CRE#1–E#1–CRE#2 and Mef2#3–CRE#3–E#4) into the luciferase reporters (Figure 3A). Myod transactivates Reporter II up to nearly 1000-fold, while that of Reporter I is less than 3-fold (Figure 3B). These data are consistent with our previous report that the E#4 box is more important for inducing *Tceal7* expression than the E#1 box [4]. Creb1 is also able to transactivate Reporter II up to 4-fold, but not Reporter I. These studies suggest that the critical motif for the transactivation by Creb1 is CRE#3 instead of CRE#1 and CRE#2 (Figure 3C). Moreover, a Creb1 mutant (Creb1-Y120F) transactivates Reporter II up to 4-fold as a wild-type control, but not Report I (Figure 3D). The enhanced transactivation with Creb1-Y120F is only present in the cases of certain, specific genes, such as somatostatin and MAP kinase phosphatase [26,43]. A further ChIP assay was performed to evaluate the interaction between Creb1 and the CRE #3 motif within the *Tceal7* promoter. As shown in Figure 3E and Figure S2, the association between Creb1 and the CRE #3 motif is confirmed. Overall, these data support the notion that the transactivation of *Tceal7* by Myod and Creb1 is mainly mediated by the E#4 and CRE#3 motifs in the distal region.

### 3.4. Transactivation of Tceal7 Expression by Myod, Mef2c and Creb1

**Figure 4:**
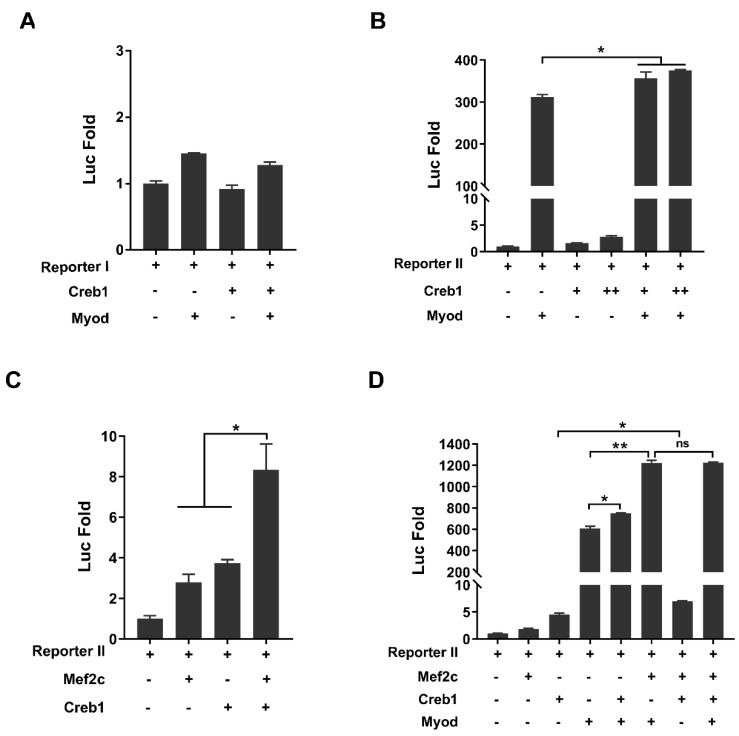
Transactivation of Reporter II by Myod, Mef2c and Creb1. (**A**) Reporter I is not responsive to Myod, Creb1 or the Myod/Creb1 complex in HEK293T cells. (**B**) Reporter II is synergistically transactivated by Myod and Creb1 up to 376-fold, while the transactivation is around 311-fold by Myod, and around 3-fold by Creb1 in HEK293T cells. (**C**) Reporter II is synergistically transactivated up to 8.3-fold, as the transactivation is 2.8-fold by Mef2c and 3.7-fold by Creb1 in HEK293T cells. (**D**) Transactivation of Reporter II by Myod, Creb1 and Mef2c. Reporter II can be transactivated synergistically by Mef2c/Creb1, Mef2c/Myod or Creb1/Myod in HEK293T cells. The transactivation of Reporter II has been saturated with all three factors: Mef2c, Creb1 and Myod. All data are shown as mean ± S.D from three replicates. * *p* < 0.05; ** *p* < 0.01 and ns, not significant.

Our above studies have demonstrated that the core element driving *Tceal7* expression is the Mef2#3–CRE#3–E#4 cluster in the distal region of the promoter. The synergistic transactivation of *Tceal7* by Myod and Mef2c has also been confirmed (Figure 2D). However, the interaction between Myod and Creb1 in transcription regulation may be collaborative or antagonistic. Herein, we examine the transcription regulation of Reporter I or Reporter II by Myod and Creb1. Reporter I is not responsive to Creb1, Myod or the Creb1/Myod complex (Figure 4A), while Reporter II is transactivated by both Myod (312-fold) and Creb1 (3-fold). Herein, we have observed a synergistic effect between Myod and Creb1 in a dose-dependent fashion (Figure 4B). We then assessed whether there is a similar synergistic effect with the Reporter II between Mef2c and Creb1. As shown in Figure 4C, there is an obvious synergy between Mef2c and Creb1, as Reporter II is transactivated up to 8.3-fold by Mef2c and Creb1, while it is transactivated up to 2.8-fold by Mef2c or 3.7-fold by Creb1 (Figure 4C). A further transcription assay with Reporter II was performed by Mef2c, Creb1 and Myod. As shown in Figure 4D, Reporter II is transactivated by Mef2c, Creb1 and Myod up to 1225-fold, comparable to that of the complex of Myod and Mef2c (1223-fold), in which the saturation of transactivation may be due to the sensibility of the reporter system, as we have observed in the synergistic transactivation between Myod and Mef2c or Creb1. Taken together, these studies suggest that *Tceal7* gene expression is regulated by Myod, Mef2c and Creb1.

### 3.5. Mef2c Interacts with Creb1

**Figure 5:**
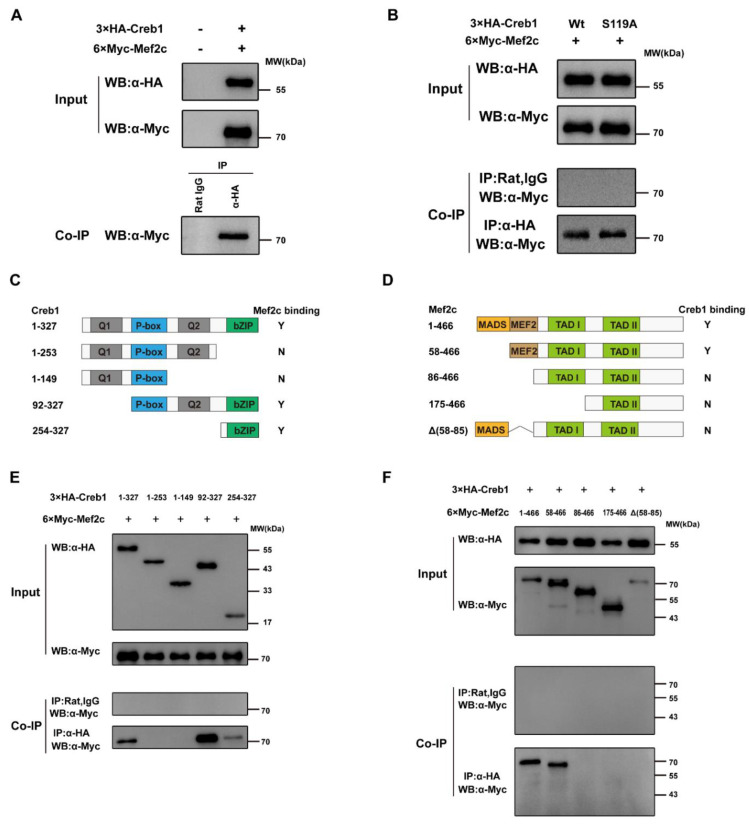
Protein–protein interaction between Mef2c and Creb1. (**A**) HEK293T cells were cotransfected 3×HA-Creb1 and 6×Myc-Mef2c for 24 h. The expression levels of Creb1 and Mef2c are detected by Western blot (WB) analysis with anti-HA or anti-Myc antibody, respectively. 6×Myc-Mef2c is coimmunoprecipitated with 3×HA-Creb1 with anti-HA antibody, but not detected in control rat IgG. (**B**) The interaction between Creb1 and Mef2c is not dependent on Creb1 S119 phosphorylation. 6×Myc-Mef2c and 3×HA-Creb1 or 3×HA-Creb1-S119A were cotransfected into HEK293T cells as the indicated combination for 24 h, followed by immunoprecipitated analysis. 6×Myc-Mef2c is coimmunoprecipitated by 3×HA-Creb1 or 3×HA-Creb1-S119A. Wt, wild-type Creb1. S119A, Creb1 Ser119 was mutated to Ala. (**C**) Schematic illustration of the interaction between Creb1 deletional constructs and Mef2c. Y, yes; N, no; Q1, glutamine-rich domain 1; P-box, phosphorylation domain; Q2, glutamine-rich domain 2; bZIP, basic region/Leu zipper domain. (**D**) Schematic summary of the interaction between Mef2c deletional constructs and Creb1. Y, yes; N, no; MADS, MADS-box domain; MEF2, MEF2 domain; TAD I, transactivation domain I; TAD II, transactivation domain II. (**E**) Analysis of the protein interaction between Cre1 deletion and Mef2c by coimmunoprecipitation (Co-IP) assays. Mef2c can only be immunoprecipitated by the deletions harboring the bZIP(254−327) domain of Creb1. (**F**) Coimmunoprecipitation (Co-IP) assays reveal that constructs containing the MEF2(58−85) domain interact with Creb1.

Neither Mef2c or Creb1 is dispensable in skeletal muscle myogenesis, but their common target genes in the skeletal muscle lineage are rarely reported. Interestingly, our above studies have revealed the synergistic transactivation between Mef2c and Creb1. These data have indicated the possibility that Mef2c and Creb1 can form a complex to regulate *Tceal7* gene expression. To validate our hypothesis, 3×HA-Creb1 and 6×Myc-Mef2c expression plasmids were constructed and co-transfected into HEK293T cells, followed by coimmunoprecipitation analysis. As shown in Figure 5A and Figures S3–S5, 6×Myc-Mef2c is coimmunoprecipitated with 3×HA-Creb1 by an anti-HA antibody, but not the control, normal Rat IgG. It is reported that a number of transcription factors, such as TOX3 and MLL [44,45], interact with the phosphorylated Creb1, but not the unphosphorylated Creb1. Herein, the Creb1-S119A mutant was constructed to examine the effect of phosphorylation of Creb1 on its association with Mef2c. Interestingly, 6×Myc-Mef2c is also detected in the coimmunoprecipitation complex of 3×HA-Creb1-S119A, as with wild-type Creb1 control (Figure 5B and Figures S6–S8). To map out the domain within Creb1 that mediates its association to Mef2c, a range of Creb1 deletional constructs were generated and analyzed (Figure 5C). Coimmunoprecipitation assays indicate that only deletions (92−327 and 254−327) harboring the bZIP domain interact with Mef2c, while deletion mutants (1−253 and 1−149) completely lose the ability to interact with Mef2c (Figure 5E and Figures S9–S12). Therefore, the bZIP domain of Creb1 is sufficient to interact with Mef2c. We then defined the Creb1-interacting surface of Mef2c using a series of deletional constructs of Mef2c (Figure 5D). Creb1 is only coimmunoprecipitated by constructs harboring the MEF2 domain, but not by other deletional constructs (86−466, 175−466 and Δ(58−85)), suggesting that the MEF2 domain is required for its interaction with Creb1 (Figure 5F and Figures S13–S16).

## 4. Discussion

**Figure 6:**
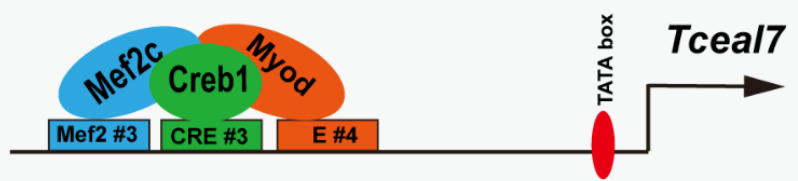
The model of transcriptional regulation of *Tceal7* in skeletal muscle. Mef2c, Creb1 and Myod form a triple complex and bind to the distal cluster region (Mef2#3, CRE#3 and E#4), thereby transactivating *Tceal7* gene expression.

We have previously reported that *Tceal7* is specifically expressed during embryonic development or skeletal muscle regeneration in a unique temporal–spatial manner, which clearly indicates the important role of *Tceal7* in the skeletal muscle lineage. We have also documented that *Tceal7* gene expression is driven by its 0.7 kb promoter [4]. Herein, we have successfully identified the key cluster of multiple motifs in the 0.7 kb promoter, Mef2#3–CRE#3–E#4; characterized the interaction between Mef2c and Creb1; and defined the essential role of the triple complex of Mef2c, Creb1 and Myod in the regulation of *Tceal7* expression (Figure 6).

The transcription activity of Creb1 is closely related to its phosphorylation status, which is dynamically regulated by protein kinases and phosphatases. We have characterized the 3.9 kb promoter of *Etv2* in the hematopoietic and endothelial lineages in previous studies [27,35,46]. Mutation of the CRE motifs, instead of the Ets motifs or NFAT-response elements, in the promoter alters the dynamic expression pattern of Etv2-ZsGreen/DR reporter in the EB system [35]. Those extensive studies have defined the essential role of the Flk1-p38-Creb1 signaling cascade in regulating the dynamic expression of *Etv2* [27,46]. The critical role of Creb1 has also been well established in the skeletal muscle lineage. Creb1 mutation results in myogenic defects during embryogenesis [47]. During skeletal muscle regeneration, Creb1 is phosphorylated and activated after acute injury [33]. Further study has revealed that Creb1 activity plays an essential role in satellite cell activation, but not cell quiescence [34]. In the present study, our investigation has characterized three conserved CRE motifs within the 0.7 kb promoter of *Tceal7* between murine and human genomes. Each of these CRE motifs plays a functional role in the promoter in the mutagenesis study (Figure 1), while only CRE#3 is pivotal for the transactivation of *Tceal7* by Creb1 in the transcription assays (Figure 3). The discrepancy may reflect a binding affinity between the Creb1 factor and the diversified CRE motifs, as the consensus CRE motif is defined as TGACGTCA. In addition, the neighbor sequence of the CRE motif may also influence the outcome of transcription activation. Taken together, CRE#3 may be the crucial motif within the *Tceal7* promoter to drive its dynamic expression pattern during skeletal muscle regeneration. However, in vivo evidence will be required for validation of this hypothesis in the future research.

Both Mef2c and Creb1 are very important cooperative factors of Myod in skeletal muscle myogenesis. The interaction between Myod and Mef2c has been well characterized as a cooperative transactivation complex, while the interaction between Myod and Creb1 may have a complicated effect on the activity of Myod in skeletal muscle myogenesis. Myod may cooperate with Creb1 to transactivate its downstream target gene, such as *Rb1*, although it has also been reported that the regulation of *Rb1* by Myod may be mediated by the enhancer of *Rb1* [6,36]. In certain circumstances, Creb1 may inhibit the activity of Myod through their interaction in the transcription regulation of the *MuSK* gene [48]. In the present study, we have observed the synergistic interaction between Myod and Creb1 with Reporter II, containing the Mef2#3–CRE#3–E#4 motif cluster. These studies suggest that the distribution of the CRE motif and the E-box in the genome may play a critical role in determining the biological significance of the interaction between Creb1 and Myod.

Our present work has also provided the first evidence of synergistic interaction between Mef2c and Creb1. Our studies have defined the interaction between Mef2c and Creb1. Interestingly, their interaction is not affected by the phosphorylation status of Creb1, indicating that p300 is not involved in the interaction between Mef2c and Creb1. The bZIP domain of Creb1 has been characterized as the DNA-binding domain, while it is also very important in the association with other proteins, such as Mesp1 and Myod [27,48]. Our present work has mapped out the interaction between the bZIP domain of Creb1 and the MEF2 domain of Mef2c. In summary, it is the bZIP domain of Creb1 interacting with Myod, Mesp1 and Mef2c; however, the outcome of these interactions may be dependent on their target gene. Further study will be required to clarify the underlying mechanisms.

Collectively, our above data suggests that Mef2c, Creb1 and Myod may form a triple complex to transactivate *Tceal7*. p38MAPK plays a very important role in skeletal muscle myogenesis and myopathy progression. Mutation of p38alpha results in a perturbed myogenic differentiation [49]. The activation of p38beta is correlated with skeletal muscle waste in the cancer patient [50,51]. p38MAPK initiates its downstream signaling through a group of substrates [52]. Creb1 is activated by S133 phosphorylation by p38MAPK, thereby recruiting the p300/CBP activator in skeletal muscle regeneration. p38MAPK-mediated phosphorylation of the transactivation domain of Mef2c could enhance its transcription activity [53]. The formation of a Myod-E47 heterodimer also relies on the phosphorylation of E47 at Ser140, mediated by p38 MAPK [54]. Interestingly, all three factors interact with the p300/CBP coactivator through their specific domains: the activation domain of Myod, the MADS domain of Mef2c and the phosphorylated S133 residue of Creb1. Therefore, p300/CBP may accelerate the recruitment of the triple complex of Mef2c, Creb1 and Myod, or stabilize their association upon p38MAPK activation during myogenic differentiation.

Mutagenesis analysis has revealed the important role of the cluster CRE#2–E#1–CRE#1 in *Tceal7* gene expression, while the transactivation of this proximal cluster by Myod or Creb1 is minimal. The discrepancy may be due to a low affinity between these factors and the transcription factor-binding motifs. Given that the cluster is adjacent to the TATA box, it is certainly possible that the proximal cluster may be important for the initiation of *Tceal7* transcription, as both Creb1 and Myod may participate in the recruitment of a pre-initiation complex [55,56]. More experiments are required to examine this hypothesis. Recently, a number of miRNAs have been reported in cancer progression through targeting human *Tceal7*, such as miR-182, miR-301a, miR-769–5p, miR-18b and miR-758-3p [57,58,59,60,61]. Taken together, more research will be required in the future to understand the complicated regulatory network of the *Tceal7* gene.

## 5. Conclusions

In the present study, we have identified a cluster of Mef2, CRE and E-box motifs in the 0.7 kb promoter of *Tceal7*. Our studies have indicated that it may be the CRE#3 motif that drives the dynamic expression of *Tceal7* during skeletal muscle regeneration. Our work has also defined the interaction between Mef2c and Creb1, providing the evidence for the triple complex of Mef2c, Creb1 and Myod. In summary, this research provides new insights into the mechanism regulating the temporal and spatial expression of the *Tceal7* gene.

## Figures and Tables

**Table 1:** Mef2, CRE and E-box motifs of the *Tceal7* promoter.

Motifs	Species	Sequence
Mef2#1	MMU	GTAAAAATAA
	HSA	CTATATTTAT
Mef2#2	MMU	CTATTTTTAA
	HSA	CTATATTTAT
Mef2#3	MMU	CTATATTTAT
	HSA	CTATATTTAT
CRE#1	MMU	TGACGTGA
	HSA	TGACGTGA
CRE#2	MMU	TGACATAA
	HSA	TGACATAA
CRE#3	MMU	TGACTTCA
	HSA	TGACTTCA
E#1	MMU	CACGTG
	HSA	CACGTG
E#4	MMU	CAGGTG
	HSA	CAGGTG

MMU, Mus musculus; HSA, Homo sapiens.

## Data Availability

Not applicable.

## Supplementary Materials

The following are available online at https://www.mdpi.com/article/10.3390/biology11030446/s1, Figure S1: ChIP assays: Determination of Mef2c binding to Mef2#3 motif within the *Tceal 7* promoter, Figure S2: ChIP assays: Determination of Creb 1 binding to CRE#3 motif within the *Tceal 7* promoter, Figure S3: Input. WB: α-HA, RAT, Figure S4: Input. WB: α-Myc, Rbt, Figure S5: Co-IP. WB: α-Myc, Rbt, Figure S6: Input. α-HA, Rat, Figure S7: Input. α-Myc, Rbt, Figure S8: Co-IP. α-Myc, Rbt, Figure S9: Input. α-HA, Rat, Figure S10: Input. WB: α-Myc, Rbt, Figure S11: IP: lgG. WB: α-Myc, Rbt, Figure S12: IP: α-HA. WB: α-Myc, Rbt, Figure S13: Input. WB: α-HA, Rat, Figure S14: Input. α-Myc, Rbt, Figure S15: IP: lgG. WB: α-Myc, Rbt, Figure S16: IP: α-HA. WB: α-Myc, Rbt.
