# Supplementary material for: Transcription Regulation of Tceal7 by the Triple Complex of Mef2c, Creb1 and Myod
Source: Biology (Basel). 2022 Mar 16;11(3):446. doi: 10.3390/biology11030446 (PMC8945367; doi:10.3390/biology11030446)
Supplement: Supplementary file 1 [file biology-11-00446-s001.zip › biology-1585908-supplementary.pdf]

Figure S1

ChIP assays : Determination of Mef2c binding to Mef2#3 motif within the *Tceal 7* promoter

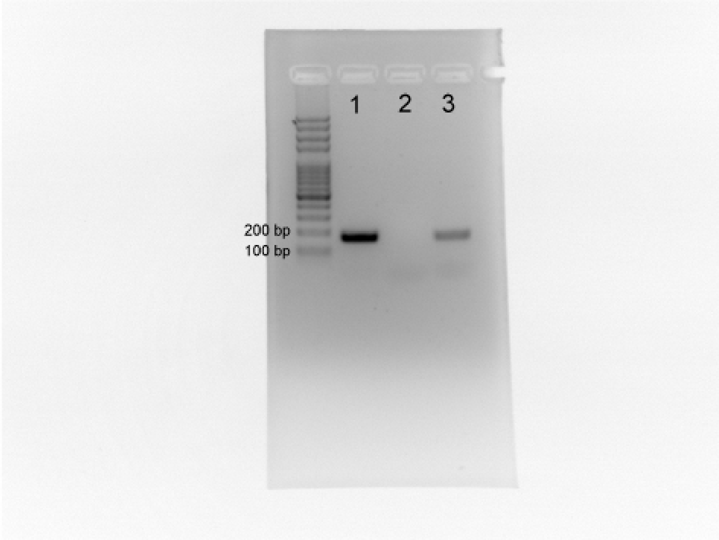

Lane 1: Input
Lane 2: IgG
Lane 3: α-HA

Figure S2

ChIP assays : Determination of Creb1 binding to CRE#3 motif within the *Tceal 7* promoter

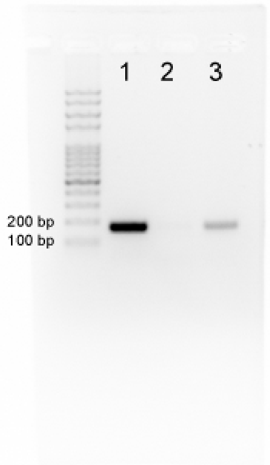

Lane 1: Input
Lane 2: IgG
Lane 3: α-HA

Figure S3：Input

WB:α-HA,Rat

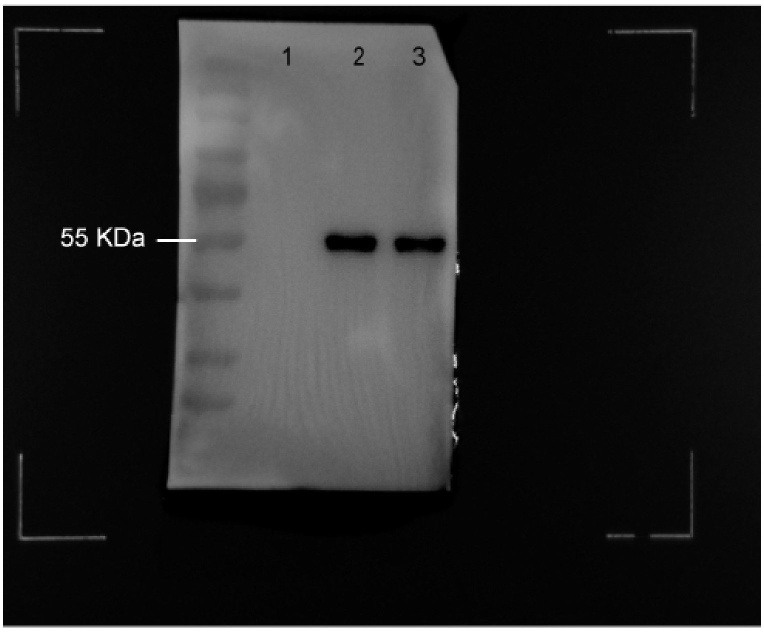

Lane 1：Untransfected cell lysate （negative control）
Lane 2：3×HA-Creb1 (sample utilized for co-IP assays)
Lane 3：3×HA-Creb1（sample utilized as the positive control）

Figure S4：Input

WB:α-Myc,Rbt

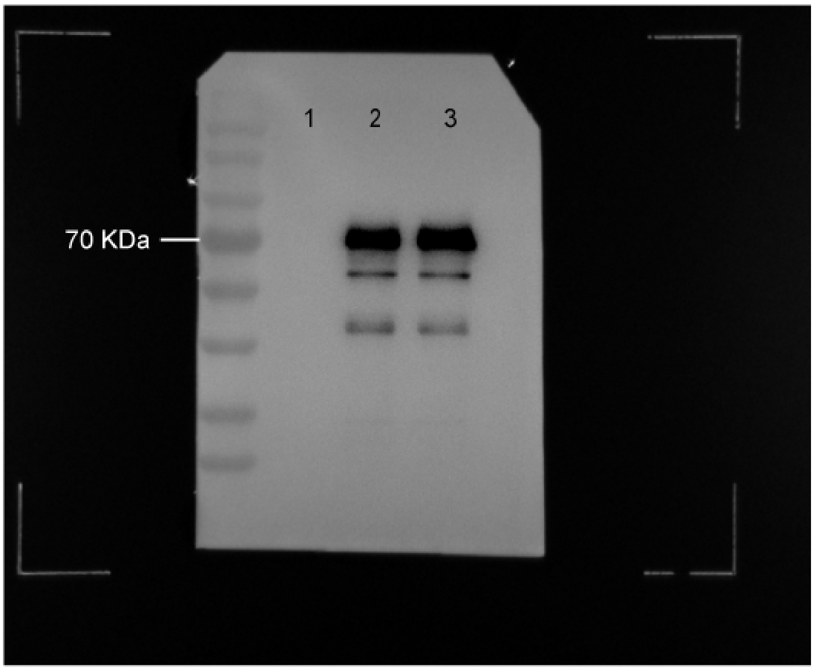

Lane 1： Untransfected cell lysate （negative control）
Lane 2： 6×Myc-Mef2c (sample utilized for co-IP assays)
Lane 3： 6×Myc-Mef2c（sample utilized as the positive control）

Figure S5：Co-IP

WB:α-Myc,Rbt

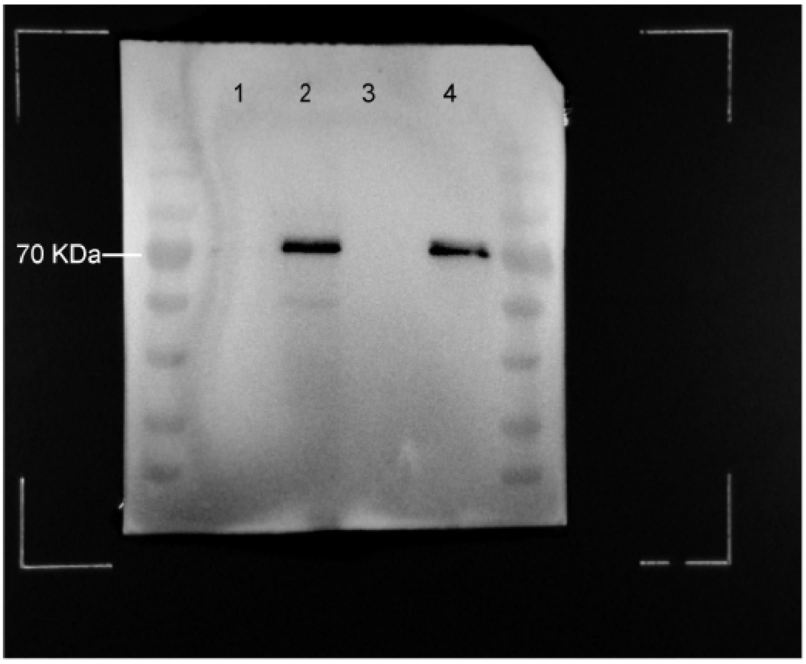

Lane 1：6×Myc-Mef2c （IP:IgG， 3×HA-Creb1）
Lane 2：6×Myc-Mef2c （IP:α-HA， 3×HA-Creb1）
Lane 3：blank
Lane 4：6×Myc-Mef2c（sample utilized as the positive control)

Figure S6：Input

WB:α-HA,Rat

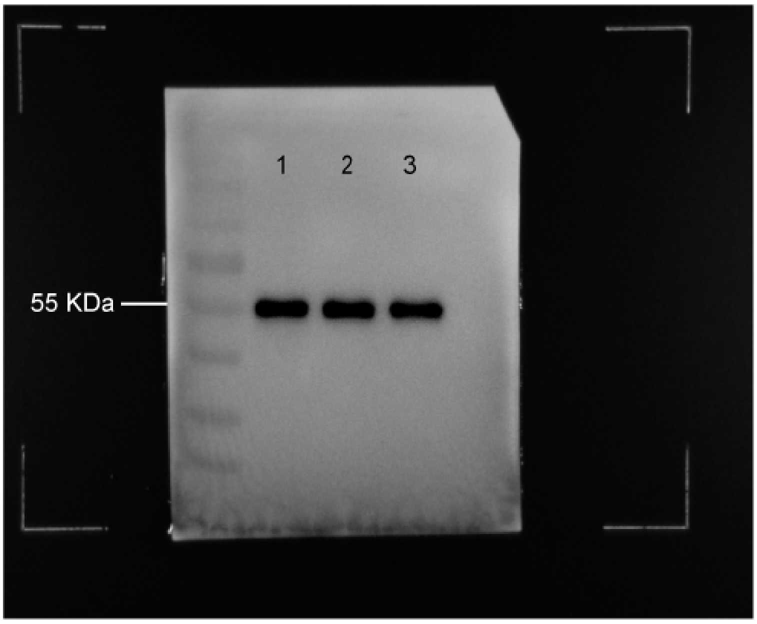

Lane 1：3×HA-Creb1-Wt (sample utilized for co-IP assays)
Lane 2：3×HA-Creb1-S119A (sample utilized for co-IP assays)
Lane 3：3×HA-Creb1-Wt （sample utilized as the positive control）

Figure S7：Input

WB:α-Myc,Rbt

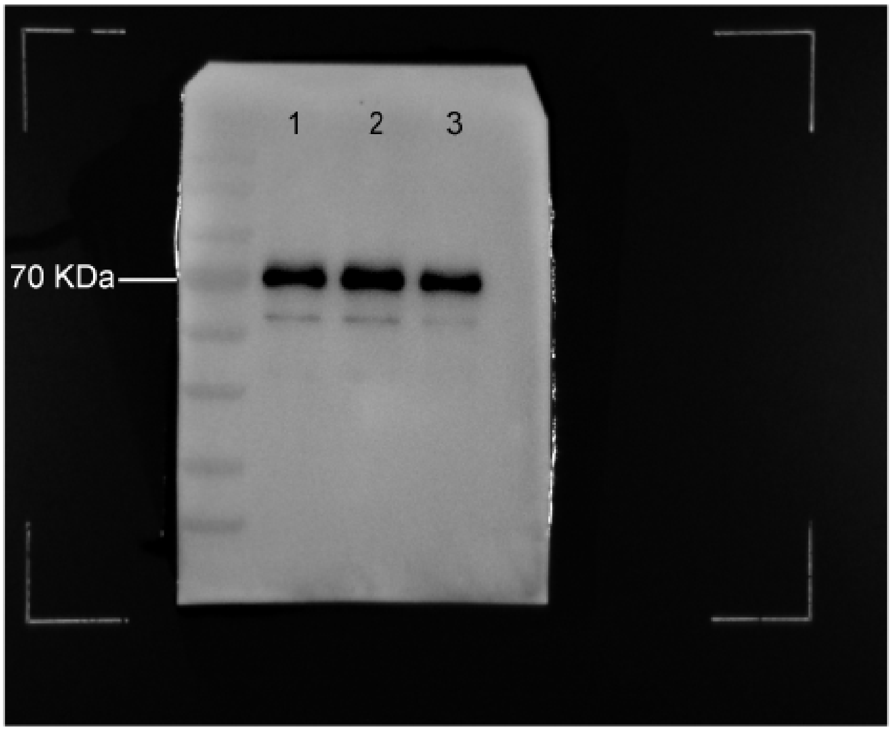

Lane 1：6×Myc-Mef2c (sample utilized for co-IP assays)
Lane 2：6×Myc-Mef2c (sample utilized for co-IP assays)
Lane 3：6×Myc-Mef2c （sample utilized as the positive control）

Figure S8：Co-IP

WB:α-Myc,Rbt

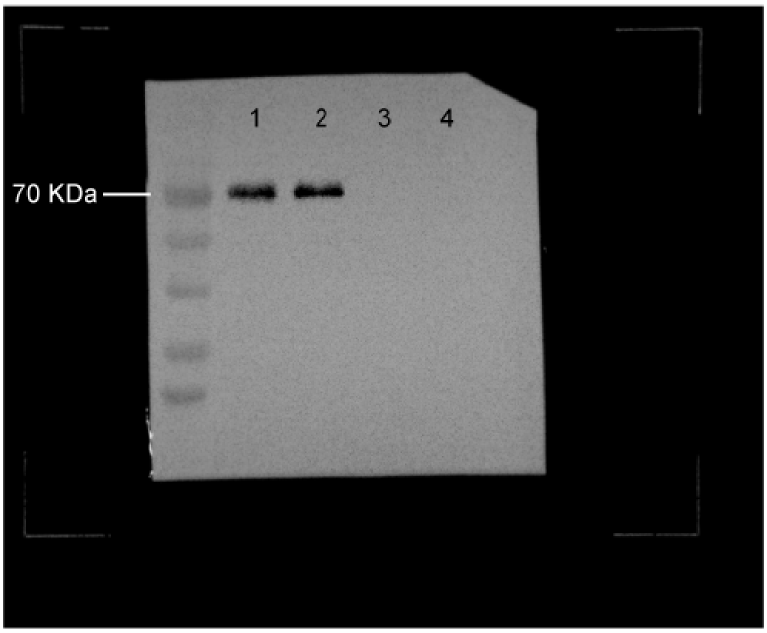

Lane 1：6×Myc-Mef2c（IP:α-HA，Wt)
Lane 2：6×Myc-Mef2c （IP:α-HA，S119A)
Lane 3：6×Myc-Mef2c（IP:IgG，Wt)
Lane 4：6×Myc-Mef2c（IP:IgG，S119A)

Figure S9： Input

WB:α-HA,Rat

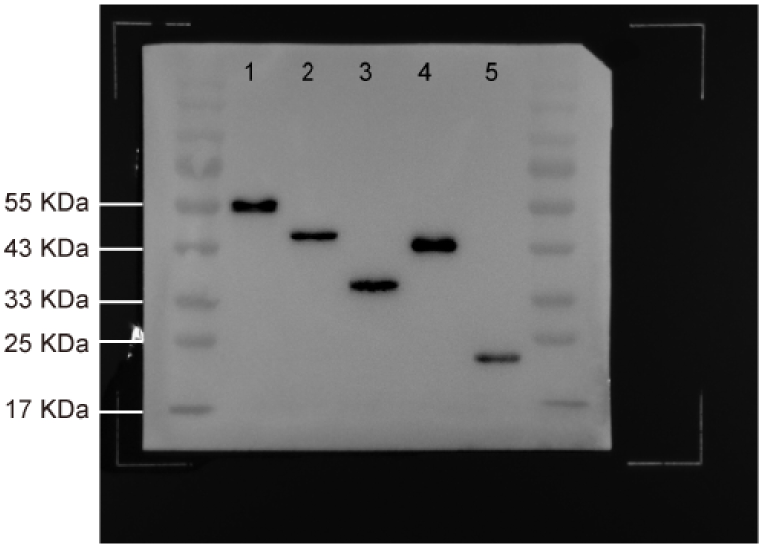

Lane 1： 3×HA-Creb1 1-327 （sample utilized for co-IP assays）
Lane 2： 3×HA-Creb1 1-253 （sample utilized for co-IP assays）
Lane 3： 3×HA-Creb1 1-149 （sample utilized for co-IP assays）
Lane 4： 3×HA-Creb1 92-327 （sample utilized for co-IP assays）
Lane 5： 3×HA-Creb1 254-327 （sample utilized for co-IP assays）

Figure S10： Input

WB:α-Myc,Rbt

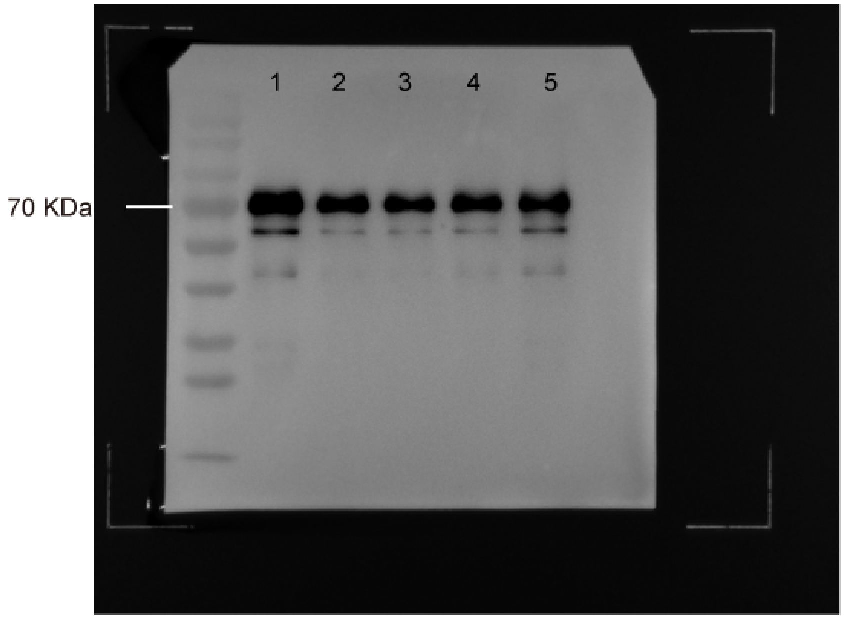

Lane 1~5 : 6×Myc-Mef2c（samples utilized for co-IP assays）

Figure S11： IP:IgG

WB:α-Myc,Rbt

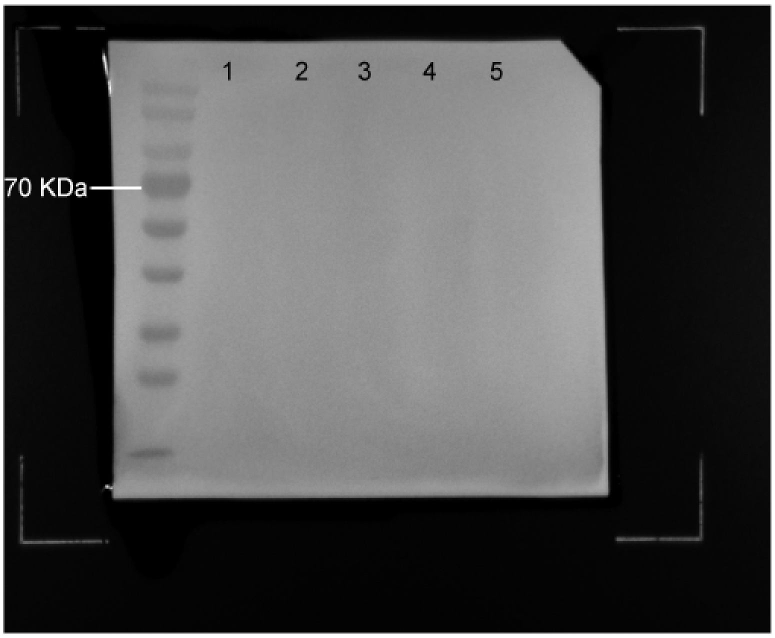

Lane 1： 6×Myc-Mef2c （IP:IgG，3×HA-Creb1 1-327）
Lane 2： 6×Myc-Mef2c （IP:IgG，3×HA-Creb1 1-253 ）
Lane 3： 6×Myc-Mef2c （IP:IgG，3×HA-Creb1 1-149 ）
Lane 4： 6×Myc-Mef2c （IP:IgG，3×HA-Creb1 92-327 ）
Lane 5： 6×Myc-Mef2c （IP:IgG，3×HA-Creb1 254-327 ）

Figure S12：IP:α-HA

WB:α-Myc,Rbt

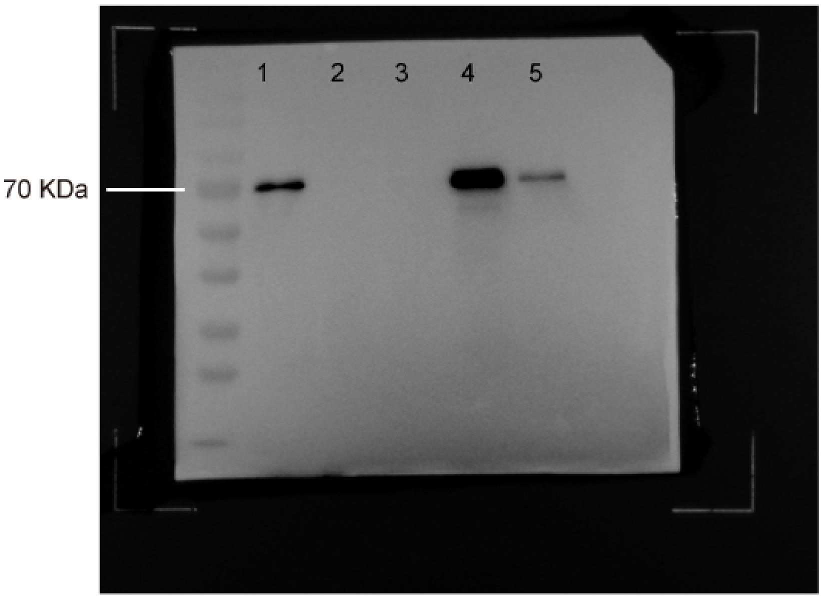

Lane 1：6×Myc-Mef2c （IP:α-HA，3×HA-Creb1 1-327）
Lane 2：6×Myc-Mef2c （IP:α-HA，3×HA-Creb1 1-253 ）
Lane 3：6×Myc-Mef2c （IP:α-HA，3×HA-Creb1 1-149 ）
Lane 4：6×Myc-Mef2c （IP:α-HA，3×HA-Creb1 92-327 ）
Lane 5：6×Myc-Mef2c （IP:α-HA，3×HA-Creb1 254-327 ）

Figure S13： Input

WB:α-HA,Rat

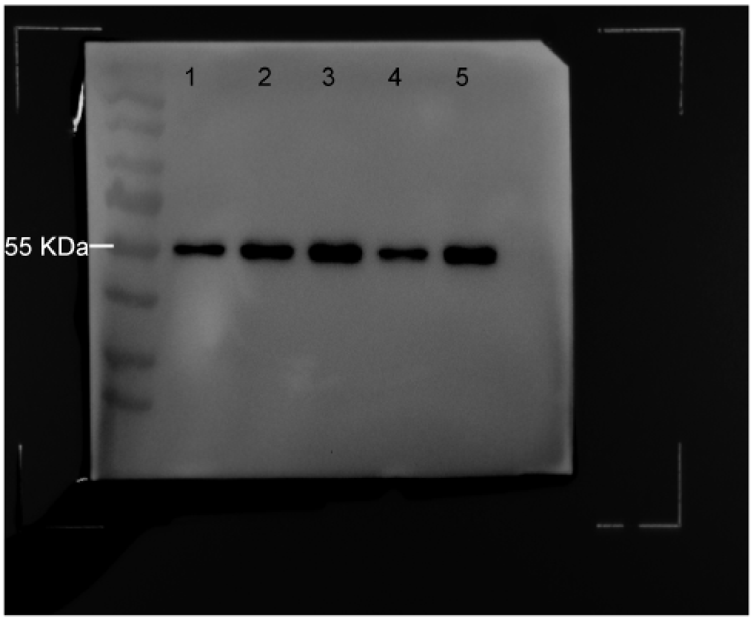

Lane 1~5 : 3×HA-Creb1（samples utilized for co-IP assays）

Figure S14: Input

WB:α-Myc,Rbt

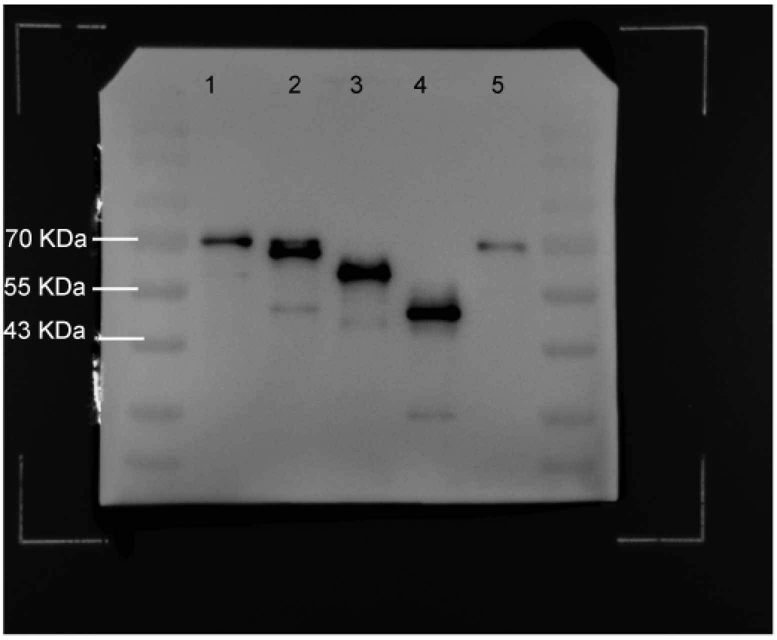

Lane 1: 6×Myc-Mef2c 1-466 (sample utilized for co-IP assays)
Lane 2: 6×Myc-Mef2c 58-466 (sample utilized for co-IP assays)
Lane 3: 6×Myc-Mef2c 86-466 (sample utilized for co-IP assays)
Lane 4: 6×Myc-Mef2c 175-466 (sample utilized for co-IP assays)
Lane 5: 6×Myc-Mef2c Δ 58-85 (sample utilized for co-IP assays)

Figure S15：IP:IgG

WB:α-Myc,Rbt

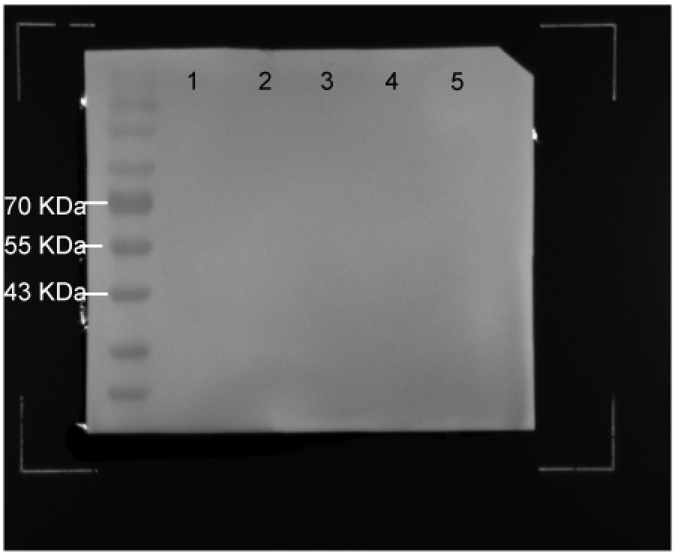

Lane 1：6×Myc-Mef2c 1-466 （IP:IgG，3×HA-Creb1）
Lane 2：6×Myc-Mef2c 58-466 （IP:IgG，3×HA-Creb1）
Lane 3：6×Myc-Mef2c 86-466 （IP:IgG，3×HA-Creb1）
Lane 4：6×Myc-Mef2c 175-466 （IP:IgG，3×HA-Creb1）
Lane 5：6×Myc-Mef2c Δ58-85 （IP:IgG，3×HA-Creb1）

Figure S16：IP:α-HA

WB:α-Myc,Rbt

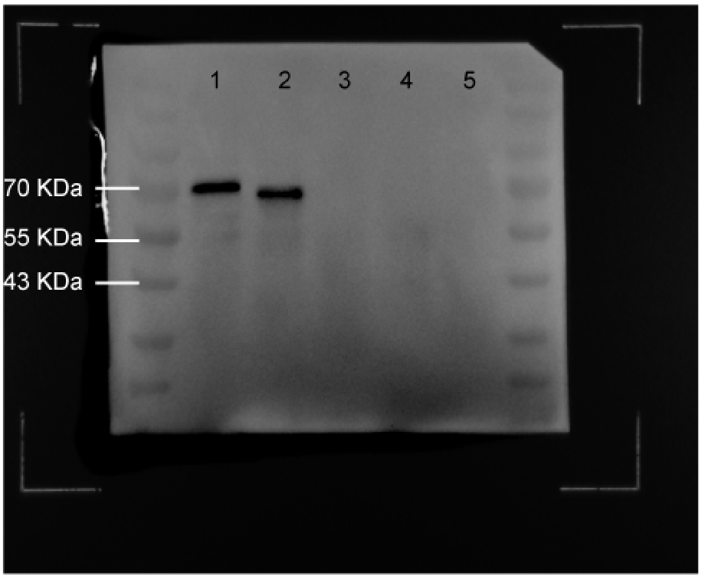

Lane 1：6×Myc-Mef2c 1-466 （IP:α-HA，3×HA-Creb1）
Lane 2：6×Myc-Mef2c 58-466 （IP:α-HA，3×HA-Creb1）
Lane 3：6×Myc-Mef2c 86-466 （IP:α-HA，3×HA-Creb1）
Lane 4：6×Myc-Mef2c 175-466 （IP:α-HA，3×HA-Creb1）
Lane 5：6×Myc-Mef2c Δ58-85 （IP:α-HA，3×HA-Creb1）
